# Effects on cardiac function, remodeling and inflammation following myocardial ischemia–reperfusion injury or unreperfused myocardial infarction in hypercholesterolemic APOE*3-Leiden mice

**DOI:** 10.1038/s41598-020-73608-w

**Published:** 2020-10-06

**Authors:** Niek J. Pluijmert, Cindy I. Bart, Wilhelmina H. Bax, Paul H. A. Quax, Douwe E. Atsma

**Affiliations:** 1 Department of Cardiology, Leiden University Medical Center, Leiden, The Netherlands; 2 Department of Surgery, Leiden University Medical Center, Leiden, The Netherlands; 3 Einthoven Laboratory for Experimental Vascular Medicine, Leiden University Medical Center, Leiden, The Netherlands

**Keywords:** Experimental models of disease, Translational research, Cardiovascular biology

## Abstract

Many novel therapies to treat myocardial infarction (MI), yielding promising results in animal models, nowadays failed in clinical trials for several reasons. The most used animal MI model is based on permanent ligation of the left anterior descending (LAD) coronary artery in healthy mice resulting in transmural MI, while in clinical practice reperfusion is usually accomplished by primary percutaneous coronary interventions (PCI) limiting myocardial damage and inducing myocardial ischemia–reperfusion (MI-R) injury. To evaluate a more similar murine MI model we compared MI-R injury to unreperfused MI in hypercholesterolemic apolipoprotein (APO)E*3-Leiden mice regarding effects on cardiac function, left ventricular (LV) remodeling and inflammation. Both MI-R and MI resulted in significant LV dilation and impaired cardiac function after 3 weeks. Although LV dilation, displayed by end-diastolic (EDV) and end-systolic volumes (ESV), and infarct size (IS) were restricted following MI-R compared to MI (respectively by 27.6% for EDV, 39.5% ESV, 36.0% IS), cardiac function was not preserved. LV-wall thinning was limited with non-transmural LV fibrosis in the MI-R group (66.7%). Two days after inducing myocardial ischemia, local leucocyte infiltration in the infarct area was decreased following MI-R compared to MI (36.6%), whereas systemic circulating monocytes were increased in both groups compared to sham (130.0% following MI-R and 120.0% after MI). Both MI-R and MI models against the background of a hypercholesterolemic phenotype appear validated experimental models, however reduced infarct size, restricted LV remodeling as well as a different distributed inflammatory response following MI-R resemble the contemporary clinical outcome regarding primary PCI more accurately which potentially provides better predictive value of experimental therapies in successive clinical trials.

## Introduction

Cardiovascular disease remains the main cause of death worldwide. Etiologically, atherosclerosis, a chronic inflammatory process, leads to the formation of lipid-rich lesions in the vascular wall, the atherosclerotic plaque. The vast majority of myocardial infarctions (MI) are a consequence of the rupture of such an inflamed atherosclerotic plaque resulting in sudden occlusion of a coronary artery^1^. Patients usually suffer from comorbidities like hypertension, hypercholesterolemia, and type 2 diabetes mellitus, amongst other risk factors of MI^2^. Current guidelines aim for timely reperfusion by primary percutaneous coronary interventions (PCI)^3^ to limit myocardial damage and reduce infarct size resulting in a better clinical outcome^4^. Paradoxically, restoration of myocardial blood flow initiates myocardial reperfusion injury by a series of events which apparently affects post-ischemic infarct healing, LV remodeling and effects of applied treatment opportunities^5,6^.

To investigate the etiology of MI and treatment opportunities, animal models are indispensable. The selection of an appropriate animal model to ensure optimal translation of novel cardioprotective therapies from bench to bedside is of the utmost importance^7–9^ which has additionally been underscored by a position paper of the European Society of Cardiology (ESC)^10^ and united in several practical guidelines with the aim of increasing rigor and reproducibility in preclinical research^11,12^. Yet until recently, most data regarding MI have been generated in animals lacking human comorbidity, spared of atherosclerosis with its associated heightened inflammatory phenotype^13^. However, a close relation has been clarified, since leukocytosis predicts cardiovascular events and precipitates plaque rupture^14^. In addition, atherosclerosis is associated with chronic monocytosis associated with increased levels of lymphocyte antigen (Ly)-6C^hi^ monocytes in response to hypercholesterolemia^15^. Following MI, an inflammatory phenotype with Ly-6C^hi^ monocytes and M1-type macrophages initially dominates which passes into a reparative phenotype with Ly-6C^lo^ monocytes and M2-type macrophages^16^, making these cells main contributors in the murine post-ischemic acute inflammatory response affecting myocardial wound healing^17^. Even though these cells are necessary in post-ischemic wound repair, excessive Ly-6C^hi^ monocytosis resulted in impaired infarct healing and accelerated deterioration of ejection fraction (EF) in unreperfused MI, underscoring the need for a balanced and coordinated response^18,19^. In addition, most animal models used for studying novel therapeutic strategies are typically characterized by permanent LAD coronary artery occlusion, whereas in contemporary clinical practice patients with acute MI receive rapid reperfusion therapy defined in the guideline as within 90 min from first medical contact with the emergency medical system till reperfusion^3^.

This scientific knowledge contributes to discussions about the relevancy of animal-derived data and whether these studies resemble the clinical setting with their human counterparts accurately which has been explicitly addressed before^12,20^. As a result, promising experimental results derived from animal studies may be viewed with some caution. This is also supported by years of experience in which numerous therapeutic strategies, for several reasons, have failed their translation into successful clinical trials, deviating from promising results in preclinical animal studies^21,22^. When using preclinical animal experiments, it is of exceptional importance to guarantee reproducibility, pay attention to cardiovascular risk factors, comorbidities, and comedications, as well as addressing long-term effects of adjunct cardioprotection after completing the entire process of scar maturation^23^. Ideally, animal models resemble the clinical setting exhibiting a human-like atherosclerotic phenotype as a major comorbidity^24^ and with respect to physiological cardiovascular parameters, in which they are exposed to temporarily myocardial ischemia followed by reperfusion experiencing human-like infarct distribution. Therefore, large animal studies remain most appropriate and are preferred over rodent models^25,26^, however, these are expensive and practically more difficult to implement. This study aims to investigate the effects of MI-R compared to unreperfused MI on cardiac function, LV remodeling and the post-ischemic inflammatory response against the background of a hypercholesterolemic phenotype in APOE*3-Leiden mice which are known to develop advanced aortic atherosclerotic lesions resembling their human counterparts when exposed to a hypercholesterolemic phenotype as a result of a cholesterol-enriched Western-type diet^27^, and to test its suitability as an experimental murine MI-R model with respect to cost effectiveness and practical ease of use.

## Results

### Plasma lipid profiles and animal characteristics

Plasma lipid profiles as expressed by total cholesterol (TC: 14.0 ± 1.2 mmol/L vs. 15.8 ± 1.1 mmol/L, *p* = 0.94) and triglyceride (TG: 1.8 ± 0.1 mmol/L vs. 1.9 ± 0.2 mmol/L, *p* = 1.00) levels did not differ between the MI-R and MI group 3 weeks after surgery (Table 1).

**Table 1:** Plasma lipid levels and animal characteristics. Plasma total cholesterol (TC), triglycerides (TG), body weight (BW), heart weight (HW), heart to body weight (HW/BW) ratio. Values are mean ± SEM. ^#^*p* < 0.05 versus MI.

	T (wk)	sham	MI-R	MI
*N*		13	15	16
TC (mmol/L)	0	17.5 ± 1.7	16.8 ± 1.3	14.8 ± 1.0
	3	13.1 ± 1.1	14.0 ± 1.2	15.8 ± 1.1
TG (mmol/L)	0	2.5 ± 0.2	2.6 ± 0.2	2.8 ± 0.1
	3	2.4 ± 0.2	1.8 ± 0.1	1.9 ± 0.2
BW (g)	0	20.7 ± 0.5	21.1 ± 0.4	20.9 ± 0.5
	3	19.6 ± 0.3	20.2 ± 0.4	20.5 ± 0.4
BW change (%)		− 4.7 ± 1.7	− 3.8 ± 0.7	− 1.6 ± 1.8
HW (mg)	3	144 ± 8	140 ± 7	167 ± 9
HW/BW ratio (mg/g)		7.3 ± 0.3	6.9 ± 0.3 ^#^	8.2 ± 0.4

Cardiac hypertrophy was assessed by determination of the heart to body weight (HW/BW) ratio. The HW/BW-ratio was significantly less increased in the MI-R group compared to the MI group (6.9 ± 0.3 mg/g vs. 8.2 ± 0.4 mg/g, *p* = 0.042), pointing to more subtle myocardial damage and limited LV remodeling and compensatory cardiac hypertrophy. Body weight (BW) was not affected between the MI and MI-R group (20.5 ± 0.4 g vs. 20.2 ± 0.4 g, *p* = 1.00) (Table 1). Surgical survival rates were 78.9% in the MI-R versus 76.2% in the MI group.

### Infarct size, cardiac remodeling and LV function

**Figure 1:**
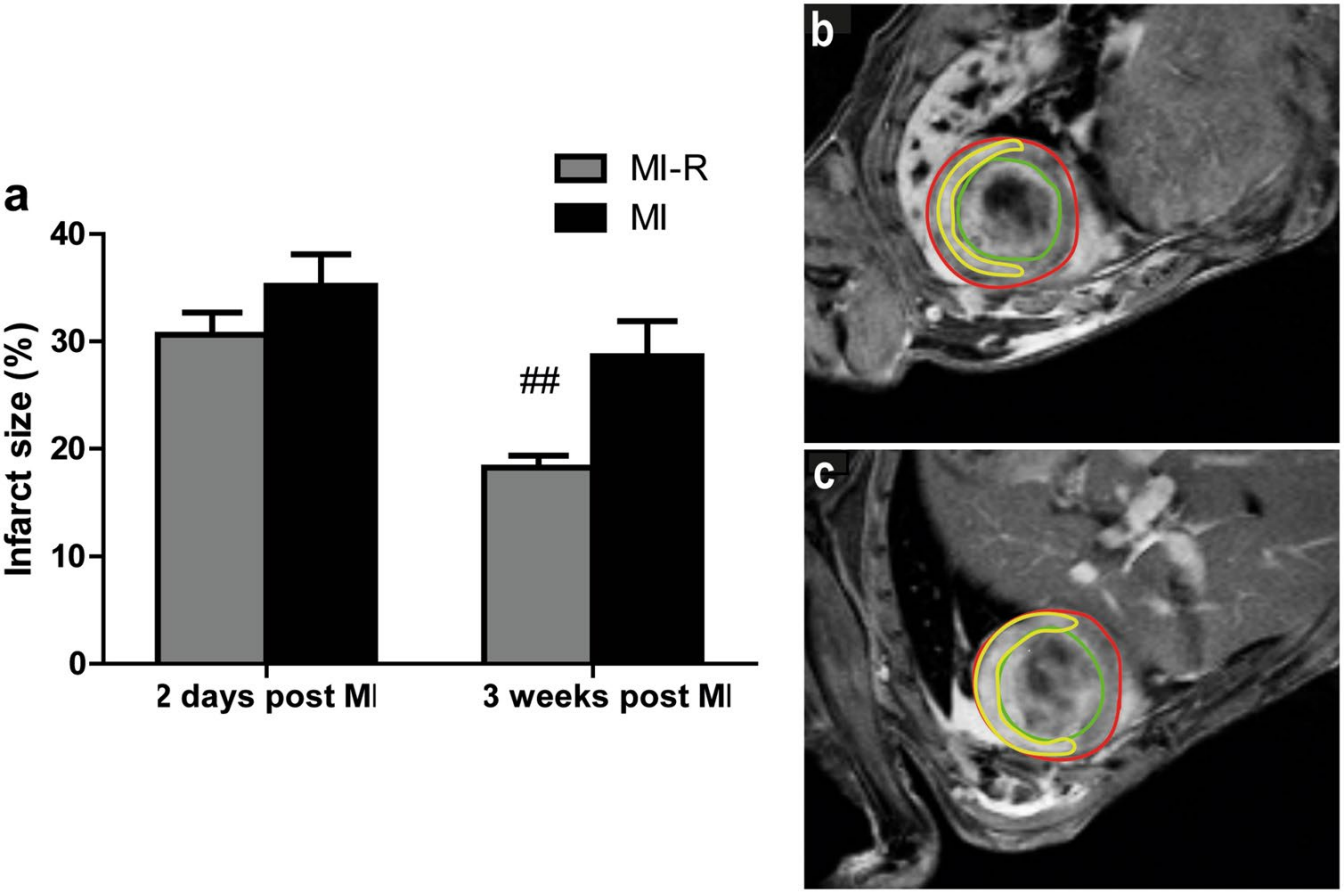
Contrast-enhanced MR imaging. After 2 days no significant difference in infarct size was observed between the MI and MI-R group. However, after 3 weeks the MI group displayed a significant increased infarct size as compared to the MI-R group (**a**; n = 15–16 per group). Typical example of contrast-enhanced MR image 2 days following non-transmural MI-R (**b**) and transmural MI (**c**). Red line indicates epicardial border, green line indicates endocardial border and yellow line indicates infarct area. Individual data points are presented in Supplemental Fig. S1. Data are mean ± SEM. ^##^*p* < 0.01 versus MI.

**Figure 2:**
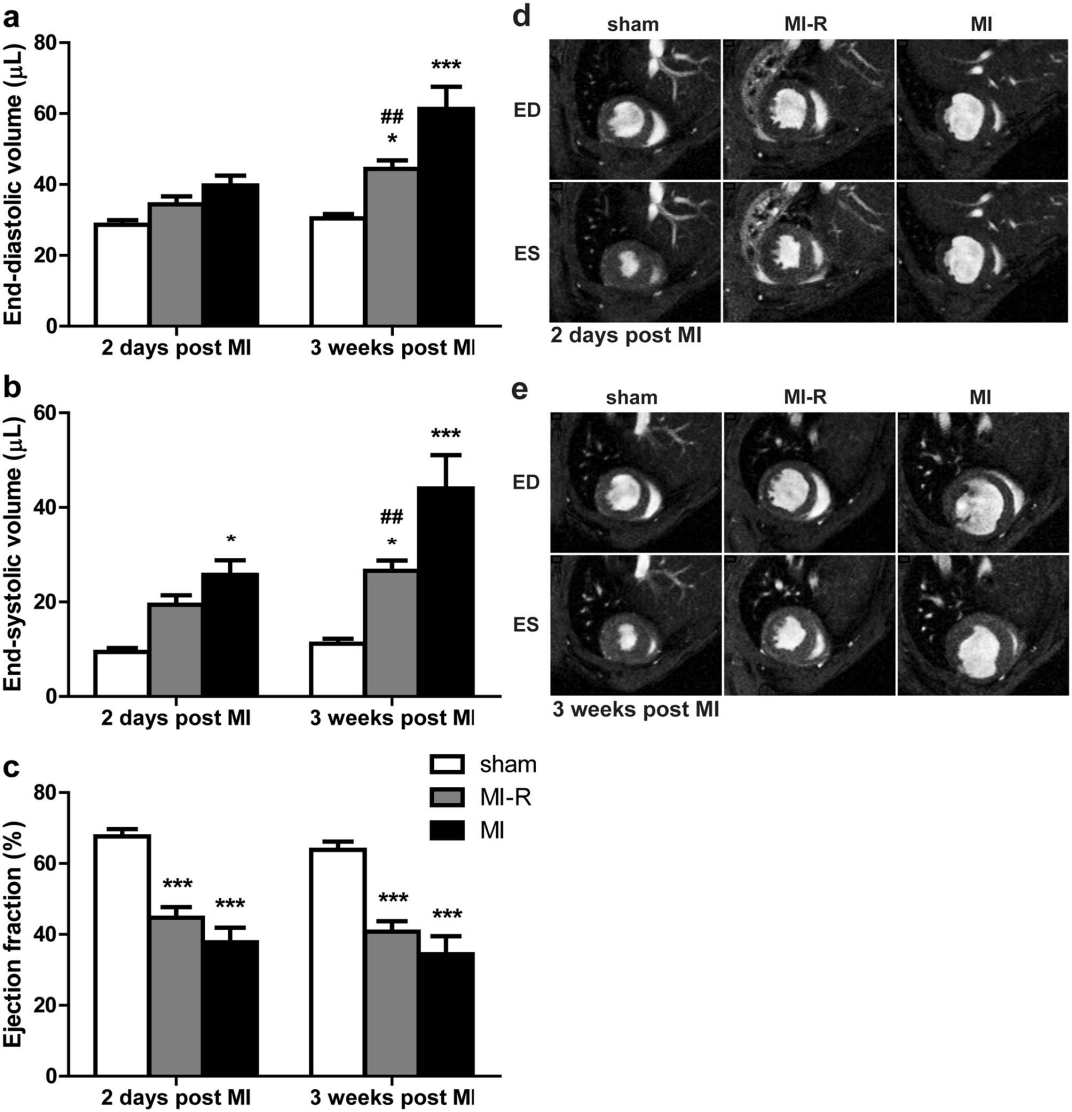
Cardiac MR imaging of LV volumes and function. Assessment of LV volumes and function after 2 days and 3 weeks (n = 13–16 per group). MI-R caused a decreased EDV (**a**) and ESV (**b**) compared to MI. EF did not significantly differ between the MI-R and MI group but was reduced in both groups as compared to sham (**c**). Typical transversal short-axis MR images at end-diastole (ED) and end-systole (ES) 2 days (**d**) and 3 weeks (**e**) after infarction in the sham, MI-R and MI groups. Individual data points are presented in Supplemental Fig. S2. Data are mean ± SEM. ^##^*p* < 0.01 versus MI; *p < 0.05, ***p < 0.001 both versus sham.

Sequential cardiac magnetic resonance imaging (MRI) after 3 weeks showed a significantly enlarged infarct size as assessed by contrast-enhanced MRI in the MI group when compared to MI-R (28.6 ± 3.3% vs. 18.3 ± 1.1%, *p* = 0.008). This effect likely resulted from augmented LV remodeling in the 3 weeks following surgery, since initial infarct size after 2 days was similar in the MI and MI-R groups (35.2 ± 2.9% vs. 30.6 ± 2.1%, *p* = 0.22; Fig. 1). Moreover, reduced scar expansion after MI-R was accompanied by limited LV dilation after 3 weeks. LV volumes, both end-diastolic volume (EDV: 44.4 ± 2.4 μl vs. 61.3 ± 6.3 μl, *p* = 0.002) and end-systolic volume (ESV: 26.6 ± 2.2 μl vs. 44.0 ± 7.0 μl, *p* = 0.003) were significantly smaller in the MI-R group compared to the MI group, consistent with limited cardiac remodeling. Again, initial LV dimensions at day 2 showed no differences between both groups (EDV, *p* = 0.81 and ESV, *p* = 0.67) which implicates impaired or at least a difference in LV remodeling during the 3 weeks following MI (Fig. 2a,b). The preserved LV volumes in the MI-R group were not associated with a significantly improved LV function, when expressed as ejection fraction (EF) following MI-R compared to unreperfused MI (40.8 ± 2.9% vs. 34.4 ± 5.1%, *p* = 0.61), which was preceded by a similar EF (*p* = 0.49) after 2 days at baseline (Fig. 2c).

### LV fibrous content and wall thickness

**Figure 3:**
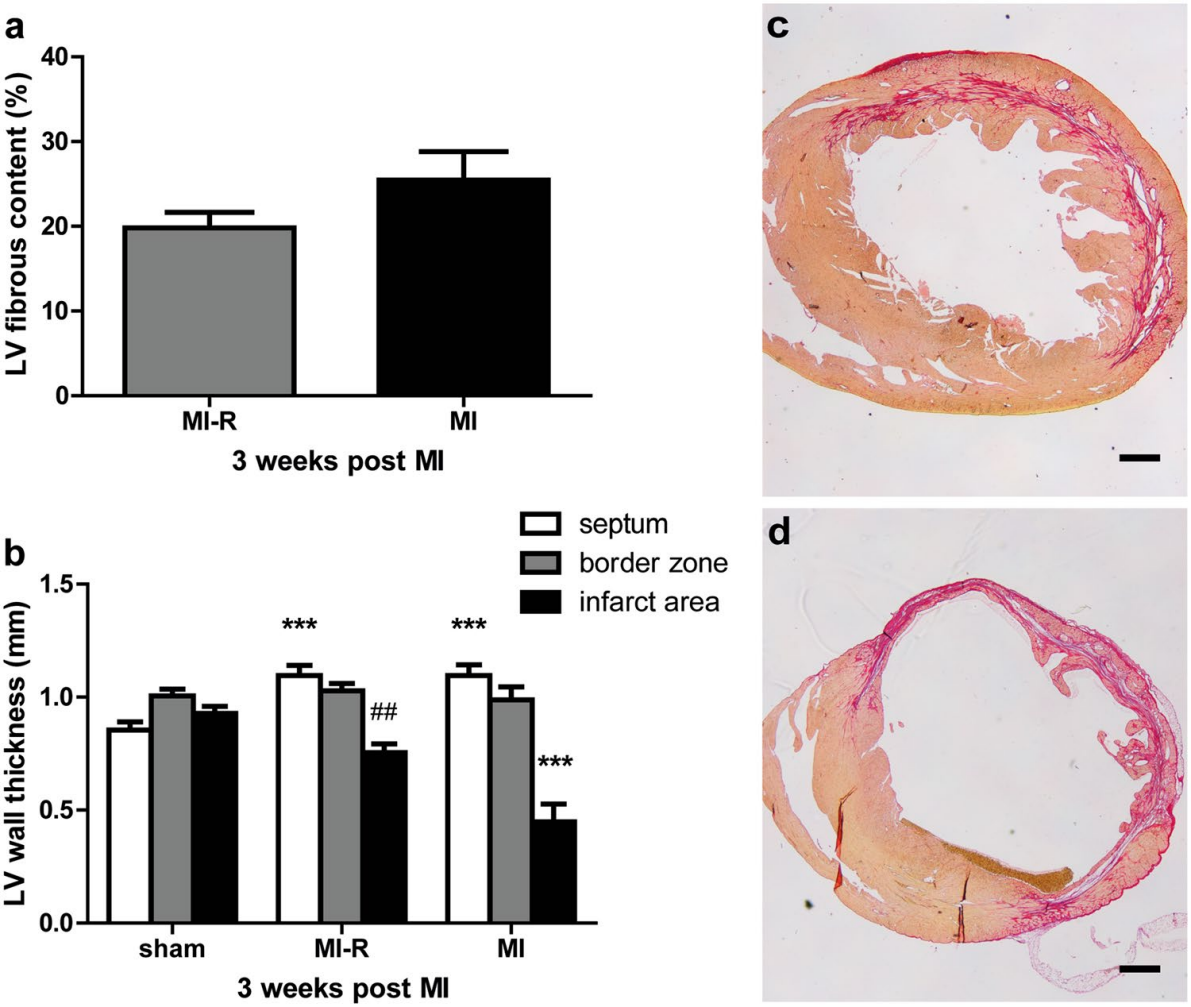
LV fibrous content and wall thickness. Histological analysis after 3 weeks (n = 9–10 per group) showed no significant difference in LV fibrous content between the MI and MI-R group (**a**). This could possibly be explained since transmural infarction in the MI group caused substantial LV wall thinning which underestimated the fibrous content as a percentage of the total LV when compared to MI-R. LV wall thickness was significantly decreased in the MI group as compared to the MI-R group (**b**). Sirius red staining of transversal short-axis sections showing typical non-transmural infarction in the MI-R (**c**) and transmural infarction with substantial LV wall thinning in the MI (**d**) group. Scale bar: 500 μm. Individual data points are presented in Supplemental Fig. S3. Data are mean ± SEM. ^##^*p* < 0.01 versus MI; ***p < 0.001 versus sham.

Histological evaluation showed no significant difference in relative LV fibrous content after 3 weeks between the MI-R and MI group (19.8 ± 1.8% vs. 25.5 ± 3.4%, *p* = 0.17, Fig. 3a). However, analysis of the LV wall thickness (Fig. 3b) demonstrated a substantial difference in distribution of fibrous content and LV wall thinning between the MI and MI-R group with a decreased LV wall thickness in the MI group compared to the MI-R group (0.45 ± 0.08 mm vs. 0.75 ± 0.04 mm, *p* = 0.001). This observation explains the non-significant difference in relative LV fibrous content since transmural infarction in the MI group caused substantial LV wall thinning (Fig. 3d) which underestimated the infarct size (IS) as a percentage of the total LV when compared to MI-R (Fig. 3c). Furthermore, infarction caused a significantly thickened interventricular septum in both the MI and MI-R group as compared to sham (1.10 ± 0.05 mm and 1.10 ± 0.04 mm vs. 0.85 ± 0.04 mm, both *p* = 0.001), indicating compensatory concentric hypertrophy (Fig. 3b).

### Systemic and local inflammatory response

**Figure 4:**
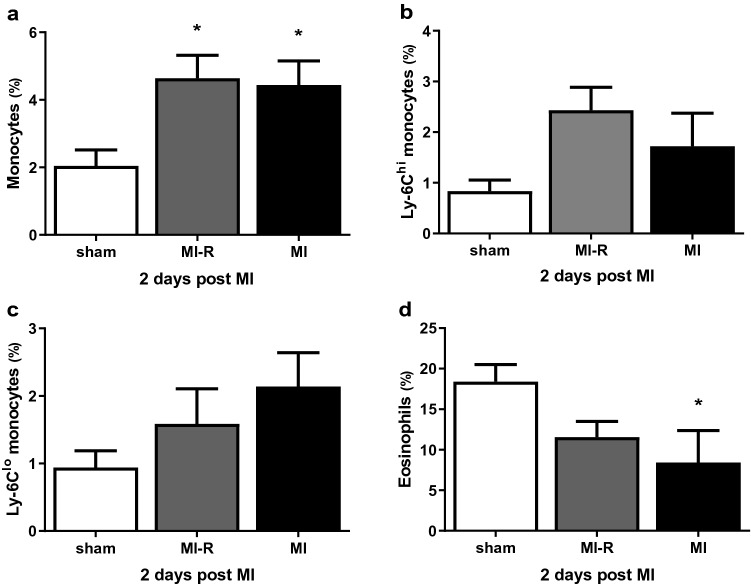
Systemic inflammatory response. FACS analysis after 2 days (n = 5–8 per group) showed increased levels of circulating monocytes in both the MI-R and MI group as compared to sham (**a**) with a different distribution pattern of Ly-6C^hi^ and Ly-6C^lo^ monocytes in both groups (**b** and **c**). Circulating eosinophils were obviously decreased in both the MI-R and MI group compared to sham (**d**). Individual data points are presented in Supplemental Fig. S4. Data are mean ± SEM. *p < 0.05 versus sham.

To unravel the systemic inflammatory response following both MI-R injury and unreperfused MI we investigated circulating inflammatory cells. Fluorescence-activated cell sorting (FACS) analysis showed significantly increased circulating monocytes (% of total leukocytes) 2 days after MI-R (4.6 ± 0.7%, *p* = 0.02) and unreperfused MI (4.4 ± 0.7%, *p* = 0.03) compared to sham (2.0 ± 0.5%; Fig. 4a). After 2 days, as compared to sham, the percentage circulating pro-inflammatory Ly-6C^hi^ monocytes (Fig. 4b) following MI-R showed a trend towards an increase (2.4 ± 0.5% vs 0.8 ± 0.2%, *p* = 0.06). On the other hand, the percentage circulating reparative Ly-6C^lo^ monocytes (Fig. 4c) showed a trend towards an increase in the MI group (2.1 ± 0.5% vs. 0.9 ± 0.2%, *p* = 0.10). Although not significantly, Ly-6C^hi^ monocytes (of total leukocytes) seems to be overrepresented 2 days after MI-R compared to MI (2.4 ± 0.5% vs. 1.7 ± 0.7%) and Ly-6C^lo^ monocytes (of total leukocytes) 2 days after MI compared to MI-R (2.1 ± 0.5% vs. 1.6 ± 0.5%). Circulating eosinophils were markedly decreased in the MI-R (11.6 ± 1.9%, *p* = 0.08) and MI (8.4 ± 3.9%, *p* < 0.05) group compared to sham (18.4 ± 2.1%) animals (Fig. 4d).

**Figure 5:**
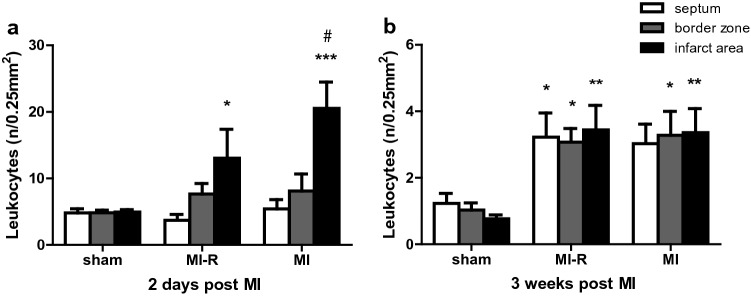
Local inflammatory response. Both MI-R and MI resulted in an increased number of infiltrated leukocytes 3 weeks after infarction as compared to sham (**b**; n = 9–10 per group). After 2 days numbers of local infiltrated leukocytes in the infarct area were higher in the MI group compared to the MI-R group (**a**; n = 5 per group). Individual data points are presented in Supplemental Fig. S5 Data are mean ± SEM. ^#^*p* < 0.05 versus MI-R; *p < 0.05, **p < 0.01, ***p < 0.001 all versus sham.

Histological analysis of the acute local inflammatory response after 2 days showed an increased local leukocyte infiltration (Fig. 5a) in the infarct area of the MI group compared to the MI-R group (20.5 ± 4.0 per 0.25 mm^2^ vs. 13.0 ± 4.3 per 0.25 mm^2^, *p* = 0.03). In addition, leukocyte infiltration compared to sham (4.9 ± 0.4 per 0.25 mm^2^) was increased in both groups (*p* < 0.001 and *p* = 0.045, respectively). Furthermore, the distribution pattern in the chronic inflammatory phase after 3 weeks differed markedly regarding a resolution of infiltrated leukocytes in general as compared to 2 days following MI. Although, a significantly increased number of local leukocyte infiltration in the border zones and infarct area was demonstrated following MI-R (3.1 ± 0.4 per 0.25 mm^2^, *p* = 0.02, and 3.4 ± 0.7 per 0.25 mm^2^, *p* = 0.002) and MI (3.3 ± 0.7 per 0.25 mm^2^, *p* = 0.01, and 3.4 ± 0.7 per 0.25 mm^2^, *p* = 0.003) as compared to sham (1.0 ± 0.2 per 0.25 mm^2^ and 0.8 ± 0.1 per 0.25 mm^2^, respectively; Fig. 5b).

## Discussion

In this study, we provide a translational murine myocardial infarction model in which we consider both a chronic inflammatory hypercholesterolemic phenotype as well as reperfusion therapy followed by scar maturation during a 3 week follow-up period, aiming to resemble the clinical setting in patients and the contemporary clinical outcome using primary PCI more accurately. Key findings are that MI-R injury caused a reduced infarct size compared to unreperfused MI, which resulted in restricted LV dilation and less LV wall thinning of the infarcted area after 3 weeks. This did not preserve cardiac function as expressed by LV ejection fraction after 3 weeks follow-up, which makes the MI-R model suitable to study hypothesized functional effects of novel therapeutic interventions. In addition, both infarction models caused an obvious local and systemic inflammatory response with subtle differences in inflammatory cell distribution illustrating different inflammatory responses following MI-R injury or unreperfused MI.

Although functional and structural long-term effects following rodent MI-R have been described before^28–30^ unreperfused MI remained the most commonly used animal model. By comparing both MI-R injury and unreperfused MI we showed the hypercholesterolemic MI-R model to be a suitable and reproducible murine infarction model to study long-term effects on cardiac function and LV remodeling following scar maturation and paying attention to specific comorbidity what may be of interest to preclinical research as indicated in recent reviews^23^ and guidelines^11,12^. This model potentially exerts better predictive value taking into account both reperfusion injury as hypercholesterolemia and their effects on post-ischemic cardiac function and remodeling, aiming for improved translation of novel cardioprotective therapies into the complex clinical reality after acute myocardial infarction including early reperfusion and a variety of comorbidities.

Cardiac MRI assessment of post-ischemic LV volumes showed significant LV dilation in both infarction models. In addition, MI-R demonstrated restricted LV dilation compared to unreperfused MI accompanied by a reduced infarct size as assessed by cardiac MRI. Both infarction models caused an impaired cardiac function, however EF was not preserved in the MI-R model as compared to unreperfused MI despite of reduced LV dilation, which is in accordance with previous reported data^28^. Thus, LV remodeling was most severe in the unreperfused MI model which was endorsed by an increased HW/BW-ratio indicating increased compensatory cardiac hypertrophy^31^.

In the discussion of translating animal-derived data towards clinical trials, the degree of infarct size is a point of interest. Beneficial effects from animal derived studies usually regards large transmural MI sizes as a result of unreperfused MI. Treatment effects are therefore often overestimated and cannot be reproduced in clinical trials where patients, who suffered a MI, exhibited an infarct size between 13 and 16% which limits the potential scope for cardioprotection^5^. This MI-R model provides an infarct size below 20% and histologically resembles the clinical setting in case of patients with timely reperfused non-transmural MI^32^, making it more suitable to predict hypothesized beneficial clinical effects in preclinical studies as endorsed by previous reported reviews, guidelines and position papers^10–12,23^. Differences in distribution of LV fibrosis and scar formation between the MI-R, with non-transmural infarction and restricted LV-wall thinning, and unreperfused MI model, with transmural infarction and extensive LV-wall thinning, explain the non-significant difference in histologically determined LV fibrous content in this study. Transmural infarction in the unreperfused MI group underestimated the LV fibrous content as a percentage of the total LV because of LV-wall thinning when compared to the non-transmural MI-R group (Fig. 3c,d). However, our results regarding infarct size and EF are in agreement with previous reported data about the infarct size to be negatively correlated with EF^33^.

Although both infarction models demonstrated a substantial post-ischemic inflammatory reaction, only subtle differences were observed regarding increased local leukocyte infiltration upon unreperfused MI in the acute inflammatory phase. This endorses the complexity and extensiveness of the post-ischemic inflammatory responses following MI-R injury and unreperfused MI which are strictly regulated. Expression of inflammatory cells, and prior cytokine and chemokine levels all depend on mutual interaction and timing of peak levels^32^, and previous studies already reported significant differences at other time points^32,34,35^. Moreover, the hypercholesterolemic phenotype as a major comorbidity^24^ influences the inflammatory response independently of myocardial ischemia, as we demonstrated before^36^.

As a result of either MI-R injury or unreperfused MI we also demonstrated locally infiltrated leukocytes and circulating monocytes to be increased and circulating eosinophil counts to be decreased as compared to non-infarcted sham controls. Our results suggest different timing and distribution patterns of the Ly-6C monocyte subsets regarding different trends of circulating pro-inflammatory Ly-6C^hi^ and reparative Ly-6C^lo^ monocytes consecutively following MI-R injury or unreperfused MI which probably affects outcome since excessive Ly-6C^hi^ monocytosis impaired infarct healing and deteriorated cardiac function^19^. The fall in circulating eosinophils has been described before in patients with an acute MI. It has been suggested that eosinophils are attracted to the site of the lesion soon after the thrombotic event in human^37^. Differences in post-ischemic inflammatory responses will definitely affect therapeutic effects when using one of both translational animal models to study cardioprotective therapies^38^ and finally influences LV remodeling in clinical trials as well^39^.

The MI-R injury model in hypercholesterolemic APOE*3-Leiden mice provides a clinically relevant translational MI model with regard to timely reperfusion. We showed limitation of LV remodeling demonstrated by a reduced infarct size, restricted LV dilation and preservation of LV wall thickness, as well as a different distributed inflammatory response following MI-R injury as compared to unreperfused MI, resembling the contemporary clinical outcome using rapid reperfusion more accurately. As well as unreperfused MI, MI-R injury still caused a significant impaired cardiac function, LV dilation and both local and systemic inflammatory response as compared to non-infarcted sham controls, making it suitable to study promising novel cardioprotective therapies in a clinically more relevant setting of rapid coronary reperfusion as we have shown before^40^.

## Methods

See the online Supplementary Information for an expanded Methods section.

### Animals and diets

All animal experiments were approved by the Institutional Committee for Animal Welfare of the Leiden University Medical Center (LUMC) and conformed to the *Guide for the Care and Use of Laboratory Animals* (NIH publication No. 85-23, revised 2011). In vivo studies were performed in hypercholesterolemic female APOE*3-Leiden mice^41^ on a semisynthetic Western-type diet supplemented with 0.4% cholesterol (AB Diets, Woerden, The Netherlands) after completing a dietary run-in period of 4 weeks at the induction of myocardial ischemia. Female rather than male APOE*3-Leiden mice were used because of their higher and stable plasma cholesterol and triglyceride levels, confined to the VLDL/LDL-sized lipoprotein fraction^42,43^. Plasma levels of TC and TG were determined after a 4-h fasting period using commercially available enzymatic kits according to the manufacturer’s protocols (11489232; Roche Diagnostics, Mannheim, Germany, and 11488872; Roche Diagnostics, Mannheim, Germany, respectively).

### Surgical myocardial infarction models

Myocardial infarction was induced with either MI-R injury or unreperfused MI model by ligation of the LAD coronary artery at day 0 in 12–14 weeks old female APOE*3-Leiden mice as described previously^28,44^. Briefly, after endotracheal intubation and ventilation mice were kept anesthetized with 1.5–2% isoflurane. Subsequently, a left thoracotomy was performed and the LAD coronary artery was ligated permanently in the MI group or during 45 min followed by permanent reperfusion in the MI-R group. Analgesia was obtained with buprenorfine s.c. pre- and post-operative. Sham operated animals were operated similarly but without ligation of the LAD (sham).

For short-term experiments, mice were euthanized 2 days after surgery to study the effects on the acute post-ischemic inflammatory response, resulting in the following groups: MI (n = 6), MI-R (n = 5), and sham (n = 8). For long-term experiments, mice were followed for 3 weeks following surgery to assess the effects on cardiac function and post-ischemic inflammation, resulting in the following groups: MI (n = 16), MI-R (n = 15), and sham (n = 13).

### Cardiac magnetic resonance imaging

LV dimensions and function were serially assessed 2 days and 3 weeks after surgery with cardiac MRI by using a 7-T MRI (Bruker Biospin, Ettlingen, Germany) equipped with a combined gradient and shim coil to obtain contrast-enhanced and cine MRI images. Initial infarct size was determined at day 2 to distinguish for any possible existing effect. Effects related to both infarction models regarding infarct size were determined by repetition of contrast-enhanced MRI after 3 weeks. LV dimensions were measured after 2 days and 3 weeks and cardiac function was determined. Image reconstruction was performed using Bruker ParaVision 5.1 software.

LV endo- and epicardial borders were delineated manually with the MR Analytical Software System (MASS) for mice (MEDIS, Leiden, The Netherlands). End-diastolic and end-systolic phases and the contrast enhanced areas were identified automatically, and the percentage of infarcted myocardium, LV-EDV, LV-ESV, LV-EF, and LV stroke volume (SV) were computed.

### Whole blood analysis

To study the systemic effects whole blood was analyzed for circulating inflammatory cells at day 2. Hematological values were obtained using a semi-automatic hematology analyzer F-820 (Sysmex; Sysmex Corporation, Etten-Leur, The Netherlands). For FACS analysis, whole blood was incubated on ice with directly conjugated antibodies directed against Ly-6C-FITC (AbD Serotec, Dusseldorf, Germany), Ly-6G-PE (BD Pharmingen, San Diego, CA, USA), CD11b-APC (BD Pharmingen, San Diego, CA, USA), CD115-PerCP (R&D Systems, Minneapolis, MN, USA), and CD45R-APC-Cy7 (eBioscience, San Diego, CA, USA).

### LV fibrous content, wall thickness, and myocardial inflammatory response

After 3 weeks mice were euthanized and blood samples were collected for analysis. Subsequently, the heart and lungs were quickly excised. Hearts were weighted as an indication of congestive heart failure and immersion-fixated and embedded in paraffin.

Sirius Red staining was used to determine LV fibrous content, as a measure of infarct size, and LV wall thickness. All measurements were performed using the ImageJ2 × 2.1.4.5 O software program (NIH, USA). For analysis of the cardiac inflammatory response sections were stained using antibodies against leukocytes (anti-CD45, 550539; BD Pharmingen, San Diego, CA, USA).

### Statistical analysis

Values were expressed as mean ± SEM. Comparisons of parameters between the MI, MI-R, and sham groups were made using 1-way analysis of variance (ANOVA) with Tukey’s correction or 2-way ANOVA with repeated measures and Tukey’s post-test in case of multiple time points. Comparisons between MI and MI-R were made using unpaired *t* tests. A value of *p* < 0.05 was considered to represent a significant difference. Statistical procedures were performed using IBM SPSS 26.0 (SPSS Inc—IBM, Armonk, NY, USA) and GraphPad Prism 8.0 (www.graphpad.com, GraphPad Software Inc, La Jolla, CA, USA) also used for the representation of figures.

## Supplementary information

Supplementary Information 1.

## Data Availability

The datasets generated during and/or analysed during the current study are available from the corresponding author on reasonable request.

## Abbreviations

APOE*3-Leiden: Apolipoprotein E3-Leiden
CO: Cardiac output
EF: Ejection fraction
HC: Hypercholesterolemic
LAD: Left anterior descending
LV: Left ventricular or left ventricle
EDV: End-diastolic volume
ESV: End-systolic volume
IS: Infarct size
Ly: Lymphocyte antigen
MI: Myocardial infarction
MI-R: Myocardial ischemia–reperfusion
MRI: Magnetic resonance imaging
NC: Normocholesterolemic
TC: Total cholesterol
TG: Triglycerides
