# Supplementary material for: Effects on cardiac function, remodeling and inflammation following myocardial ischemia–reperfusion injury or unreperfused myocardial infarction in hypercholesterolemic APOE*3-Leiden mice
Source: Sci Rep. 2020 Oct 6;10:16601. doi: 10.1038/s41598-020-73608-w (PMC7538581; doi:10.1038/s41598-020-73608-w)
Supplement: Supplementary file 1 — Supplementary Information 1. [file 41598_2020_73608_MOESM1_ESM.docx]

**Supplementary Information**

**Effects on cardiac function, remodeling and inflammation following myocardial ischemia-reperfusion injury or unreperfused myocardial infarction in hypercholesterolemic APOE*3-Leiden mice**

Niek J. Pluijmert^1*^, Cindy I. Bart^1^, Wilhelmina H. Bax^1^, Paul H.A. Quax ^2,3^, Douwe E. Atsma^1^

**Supplementary figures with legends**

This concerns all figures from the manuscript presented here as individual data points.

**Supplementary Fig. S1**

**Supplementary Fig. S1: Contrast-enhanced MR imaging.** Infarct size after two days and three weeks in the MI-R and MI group as assessed by MRI (n=15-16 per group). Data are mean±SEM. ##p<0.01 vs. MI.

**Supplementary Fig. S2**

**Supplementary Fig. S2: Cardiac MR imaging of LV volumes and function.** Assessment of LV volumes (**a** and **b**) and function (**c**) after two days and three weeks in the sham, MI-R and MI group (n=13-16 per group). Data are mean±SEM. ##p<0.01 vs. MI; *p<0.05, ***p<0.001 both vs. sham.

**Supplementary Fig. S3**

**Supplementary Fig. S3: LV fibrous content and wall thickness.** Histological analysis after three weeks of LV fibrous content (**a**) and LV wall thickness (**b**) in the sham, MI-R and MI group (n=9-10 per group). Data are mean±SEM. ##p<0.01 vs. MI; ***p<0.001 vs. sham.

**Supplementary Fig. S4**

******

**Supplementary Fig. S4: Systemic inflammatory response.** FACS analysis after two days (n=5-8 per group) with levels of circulating monocytes (**a**), Ly-6C^hi^ (**b**) and Ly-6C^lo^ (**c**) monocytes, and eosinophils (**d**) in the sham, MI-R and MI group. Data are mean±SEM. *p<0.05 vs. sham.

**Supplementary Fig. S5**

******

**Supplementary Fig. S5: Local inflammatory response.** Local infiltration of leukocytes in the septum, border zone and infarct area after two days (**a**; n=4-5 per group) and three weeks (**b**; n=9-10 per group) in the sham, MI-R and MI group. Data are mean±SEM. #p<0.05 vs. MI; *p<0.05, **p<0.01, ***p<0.001 all vs. sham.

**Supplementary Methods – expanded version**

**Animals and diets**

All animal experiments were approved by the Institutional Committee for Animal Welfare of the Leiden University Medical Center (LUMC) and conformed to the *Guide for the Care and Use of Laboratory Animals* (NIH publication No. 85-23, revised 2011). Transgenic female APOE*3-Leiden mice^35^, backcrossed for more than 40 generations on a C57Bl/6J background (bred in the animal facility of the LUMC), aged 8-10 weeks at the start of a dietary run-in period were used for this experiment. Mice were fed a semisynthetic Western-type diet supplemented with 0.4% cholesterol (AB Diets, Woerden, The Netherlands) 4 weeks prior to surgery, earlier proven to attain a stable hypercholesterolemic phenotype, and was continued throughout the complete experiment. Female rather than male APOE*3-Leiden mice were used because of their higher and stable plasma cholesterol and triglyceride levels, confined to the VLDL/LDL-sized lipoprotein fraction^36,37^. Mice were housed under standard conditions in conventional cages and received food and water ad libitum.

**Plasma lipid analysis**

Plasma levels of total cholesterol (TC) and triglycerides (TG) were determined for randomization one week before surgery and at the end of the experiment. After a 4-hour fasting period, plasma was obtained via tail vein bleeding (~50μL) and assayed for total cholesterol (TC) and triglycerides (TG) levels using commercially available enzymatic kits according to the manufacturer’s protocols (11489232; Roche Diagnostics, Mannheim, Germany, and 11488872; Roche Diagnostics, Mannheim, Germany, respectively).

**Surgical myocardial infarction models**

Myocardial infarction was induced with either a MI-R injury or an unreperfused MI model by ligation of the left anterior descending (LAD) coronary artery at day 0 in 12-14 weeks old female APOE*3-Leiden mice as described previously^23,38^. Briefly, mice were pre-anesthetized with 5% isoflurane in a gas mixture of oxygen and room air and placed in a supine position on a heating pad (37°C). After endotracheal intubation and ventilation (rate 160 breaths/min, stroke volume 190μL; Harvard Apparatus, Holliston, MA, USA), mice were kept anesthetized with 1.5-2% isoflurane. Subsequently, a left thoracotomy was performed in the 4^th^ intercostal space and the left anterior descending (LAD) coronary artery was ligated using a 7-0 prolene suture permanently in the MI group or during 45 minutes, knotted on a 2mm section of a plastic tube to protect the myocardium when removed, followed by permanent reperfusion in the MI-R group. Ischemia was confirmed by myocardial blanching. During this period muscle flaps were folded back and covered with a pre-warmed wet surgical mesh. Body temperature was kept constant between 35-37°C. In the MI-R group, mice received an intraperitoneal injection of lidocain (6mg/kg) after 35 minutes of ischemia to prevent cardiac arrhythmias caused by reperfusion^39^, and after 45 minutes of ischemia, permanent reperfusion was established. Subsequently, the thorax was closed in layers with 5-0 prolene suture and mice were allowed to recover. Analgesia was obtained with buprenorfine s.c. (0.1mg/kg) pre-operative and 10-12h post-operative. Sham operated animals were operated similarly but without ligation of the LAD (sham).

**Experimental groups**

For short-term experiments, mice were exposed to MI or MI-R and euthanized two days after surgery to study the effects on the acute post-ischemic inflammatory response. The following groups were included in this study: MI (n=6), MI-R (n=5), and sham (n=8).

For long-term experiments, per group (n=20) mice were exposed to MI or MI-R and followed for three weeks to assess the effects on cardiac function and post-ischemic inflammation. As a result of mortality related to the invasive procedure and exclusion of non-infarcted mice (failed MI procedure) the following groups were included in this study: MI (n=16), MI-R (n=15), and sham (n=13).

**Short-term experiments**

**Whole blood analysis**

To study the systemic effects whole blood was analyzed for monocytosis at day 2. Hematological values obtained were white blood cell counts (WBC, x10^6^/mL), red blood cell counts (RBC, x10^9^/mL), and platelets (PLT, x10^6^/mL) using a semi-automatic hematology analyzer F-820 (Sysmex; Sysmex Corporation, Etten-Leur, The Netherlands). For FACS analysis, 35μL of whole blood was incubated for 30 min on ice with directly conjugated antibodies directed against Ly-6C-FITC (AbD Serotec, Dusseldorf, Germany), Ly-6G-PE (BD Pharmingen, San Diego, CA, USA), CD11b-APC (BD Pharmingen, San Diego, CA, USA), CD115-PerCP (R&D Systems, Minneapolis, MN, USA), and CD45R-APC-Cy7 (eBioscience, San Diego, CA, USA). To identify thresholds for lineage-positivity 35μL whole blood was incubated with an appropriate cocktail of isotype controls.

**Myocardial inflammatory response**

For analysis of the local inflammatory response, paraffin-embedded hearts (n=5 each group) were cut into serial transverse sections of 5μm along the entire long-axis of the LV and mounted on slides. Paraffin sections of the mid-infarct region of the heart were stained using antibodies against leukocytes (anti-CD45, 550539; BD Pharmingen, San Diego, CA, USA). The number of leukocytes was expressed as a number per 0.25mm^2^ in the septum (2 areas), border zones (2 areas), and infarcted myocardium (3 areas).

**Long-term experiments**

**Cardiac magnetic resonance imaging**

Cardiac parameters were assessed two days and three weeks post MI using a 7-Tesla MRI (Bruker Biospin, Ettlingen, Germany) equipped with a combined gradient and shim coil, which is inserted into the magnet bore. Mice were pre-anesthetized as described above and kept anesthetized with 1.5-2% isoflurane. Respiratory rate was monitored by a respiration detection cushion, which was placed underneath the thorax and connected to a gating module to monitor respiratory rate (SA Instruments, Inc., Stony Brook, NY). Image reconstruction was performed using Bruker ParaVision 5.1 software.

***Infarct size***

To determine infarct size, contrast enhanced MR imaging was performed after injection of a 150µL bolus (0.5mmol/ml) of gadolinium-DPTA (Gd-DPTA, Dotarem, Guerbet, the Netherlands) via the tail vein*.*A gradient echo sequence (FLASH) was used to acquire a set of 14 contiguous 0.7mm contract-enhanced slices in short-axis orientation covering the entire heart. Imaging parameters were: Imaging parameters were: echo time of 1.9ms, repetition time of 84.16ms, field of view (33mm^2^), and a matrix size of 192x256.

***Left ventricular function***

Assessment of cardiac function was performed with a high-resolution 2D FLASH cine sequence to acquire a set of 9 contiguous 1mm slices in short-axis orientation covering the entire heart. Imaging parameters were: echo time of 1.49ms, repetition time of 5.16ms, field of view (26mm^2^), and a matrix size of 144x192.

***Image analysis***

All MR image data was analysed with the MASS for mice software package (MEDIS, Leiden, the Netherlands). The endocardial and epicardial borders were manually delineated and a reference point was positioned by an investigator blinded to treatment. Subsequently, the infarcted area of the LV, end-diastolic volume (EDV), end-systolic volume (ESV), ejection fraction (EF), stroke volume (SV), cardiac output (CO), ED wall thickness, ES wall thickness, wall thickening and wall motion were computed automatically.

After three weeks mice were euthanized and blood samples were collected for analysis. Subsequently, the heart and lungs were quickly excised. Hearts were weighted as an indication of congestive heart failure and immersion-fixated in 4% paraformaldehyde for 24 hours and embedded in paraffin.

**LV fibrous content and wall thickness**

After selecting 10 mice of the mid-population of each experimental group, by using the functional MRI data after three weeks, paraffin-embedded hearts (n=10 each group) were cut into serial transverse sections of 5μm along the entire long-axis of the LV and mounted on slides. To analyze collagen deposition as an indicator of the fibrotic area, every 50^th^ section of each heart was stained with Sirius Red. LV fibrous content as a measure of infarct size was determined by planimetric measurement of all sections and calculated as fibrotic area divided by the total LV wall surface area including the interventricular septum.

LV wall thickness was measured in five different sections centralized in the infarct area. Per section, wall thickness was analyzed at three places equally distributed in the infarcted area, both border zones, and two places of the interventricular septum. Measurements were performed perpendicular to the ventricular wall. Corresponding areas were used for measurements in the non-infarcted sham group. All measurements were performed by an observer blinded to the groups, using the ImageJ2x 2.1.4.5 O software program (NIH, USA).

**Myocardial inflammatory response**

For analysis of the cardiac inflammatory response paraffin sections of the mid-infarct region of the heart were stained using antibodies against leukocytes (anti-CD45, 550539; BD Pharmingen, San Diego, CA, USA). The number of leukocytes was expressed as a number per 0.25mm^2^ in the septum (2 areas), border zones (2 areas), and infarcted myocardium (3 areas).

**Statistical analysis**

Values were expressed as mean ± SEM. Comparisons of parameters between the MI, MI-R, and sham groups were made using 1-way analysis of variance (ANOVA) with Tukey’s correction or 2-way ANOVA with repeated measures and Tukey’s post-test in case of multiple time points. Comparisons between MI and MI-R were made using unpaired *t*-tests. A value of p<0.05 was considered to represent a significant difference. Statistical procedures were performed using IBM SPSS 26.0 (SPSS Inc – IBM, Armonk, NY, USA) and GraphPad Prism 8.0 (GraphPad Software Inc, La Jolla, CA, USA) also used for the representation of figures.
